# Gastro-tracheal fistula following esophageal cancer surgery through the retrosternal route: a case report

**DOI:** 10.1186/s40792-024-02052-z

**Published:** 2024-11-04

**Authors:** Seitaro Nishimura, Kazuhiro Noma, Kento Kawasaki, Masashi Hashimoto, Takuya Kato, Naoaki Maeda, Shunsuke Tanabe, Yasuhiro Shirakawa, Toshiyoshi Fujiwara

**Affiliations:** 1 Department of Gastroenterological Surgery, Dentistry and Pharmaceutical Sciences, Okayama University Graduate School of Medicine, 2-5-1 Shikata-Cho, Kita-Ku, Okayama, 700-8558 Japan; 2 Department of Surgery, Hiroshima City Hiroshima Citizens Hospital, 7-33 Motomachi, Naka-Ku, Hiroshima, Japan

**Keywords:** Gastro-tracheal fistula, Esophageal cancer, Retrosternal route, Esophageal surgery

## Abstract

**Background:**

Gastro-tracheal fistula is a rare but serious complication after esophageal surgery, often requiring long-term treatment and invasive procedures. Gastro-tracheal fistula usually occurs through the posterior mediastinal route and rarely through the retrosternal route. No previous reports have described gastro-tracheal fistula after retrosternal route reconstruction was cured by conservative treatment.

**Case presentation:**

A 70-year-old man with lower thoracic esophageal cancer underwent thoracoscopic esophagectomy in the prone position and gastric tube reconstruction through the retrosternal route with neck anastomosis after neoadjuvant chemotherapy. Despite anastomotic leakage on postoperative day 10, his general condition was stable, and he was managed conservatively with antibiotics and gastric tube decompression. On day 29, he presented with high fever and a gastro-tracheal fistula was observed by esophagography. Conservative management was continued because the patient remained stable. On day 48, esophagography showed that the fistula was undetectable. The patient was able to take fluids orally. He progressed well on an oral diet and was transferred to a different hospital.

**Conclusions:**

A gastro-tracheal fistula, although rare, can occur after retrosternal route reconstruction. When a patient is stable, gastro-tracheal fistula after retrosternal route reconstruction may be cured by conservative treatment.

## Background

Gastro-tracheal fistula (GTF) is a rare but serious complication of esophageal surgery. Rectification of GTF often requires long-term treatment and invasive procedures. Most previous reports of GTF describe the occurrence of the fistula through the posterior mediastinal route. The reason is that fistulae formation involves direct communication between the anastomosis site and adjacent trachea or main bronchus.

Here, we report a case of GTF after esophageal surgery through the retrosternal route, successfully repaired with conservative therapy.

## Case presentation

A 70-year-old man was admitted to our hospital with esophageal squamous cell carcinoma. The preoperative diagnosis was clinical T3N2M0 stage III (Japanese classification of esophageal cancer, 10th edition). After two courses of preoperative chemotherapy, he underwent thoracoscopic esophagectomy in the prone position, gastric tube reconstruction through the retrosternal route with neck anastomosis, three-field lymph node dissection, enterostomy, and cholecystectomy.

**Figure. 1:**
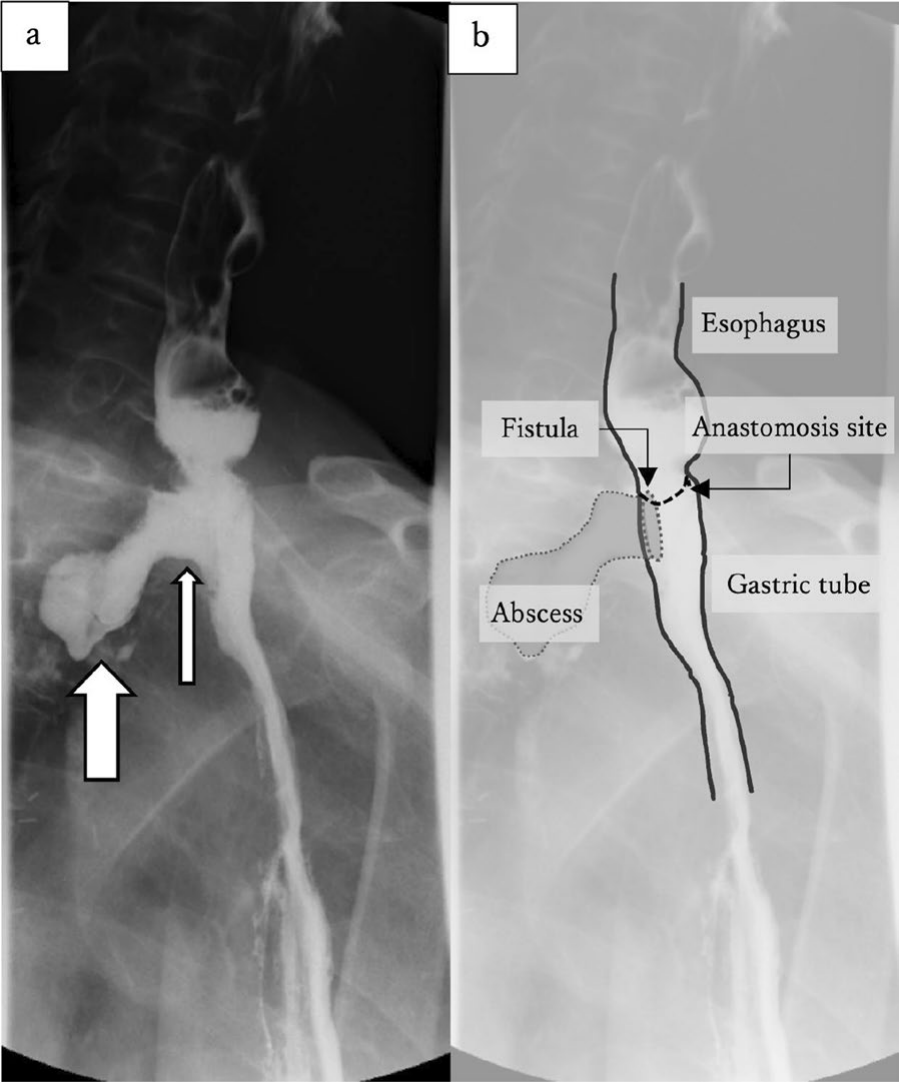
Esophagography. **a** A fistula (thin arrow) can be detected from the posterior anastomotic wall and contrast medium is present in mediastinal space (thick arrow). **b** The illustration corresponds to **a**

On postoperative day 10, we performed esophagography to check the anastomosis site. We observed anastomotic leakage and a fistula from the posterior anastomotic wall to the mediastinal abscess (Fig. 1a and b). His general condition was stable and could be managed conservatively with antibiotics and gastric tube decompression.

**Figure. 2:**
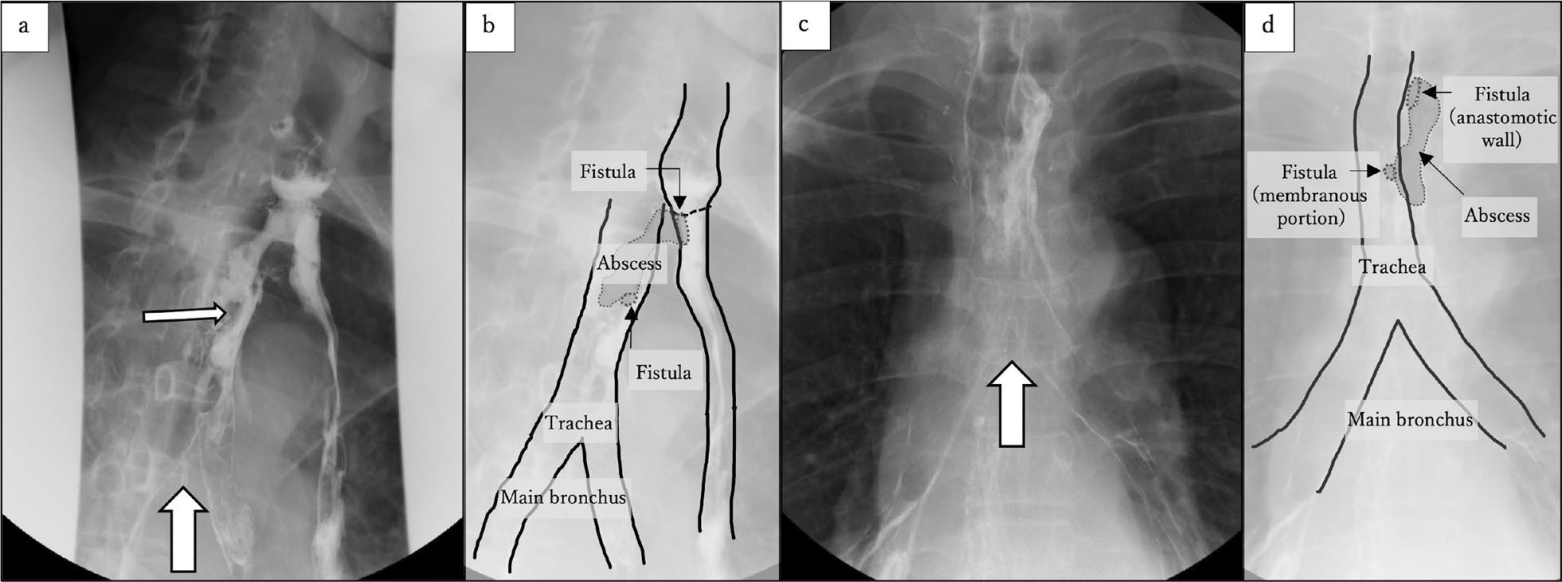
Esophagography. The carina of trachea (thick arrow). **a** A fistula (thin arrow) can be detected from the posterior anastomotic wall to the trachea. **b** The illustration corresponds to a. **c** Contrast medium is present in the trachea and main bronchus. d The illustration corresponds to c

**Figure. 3:**
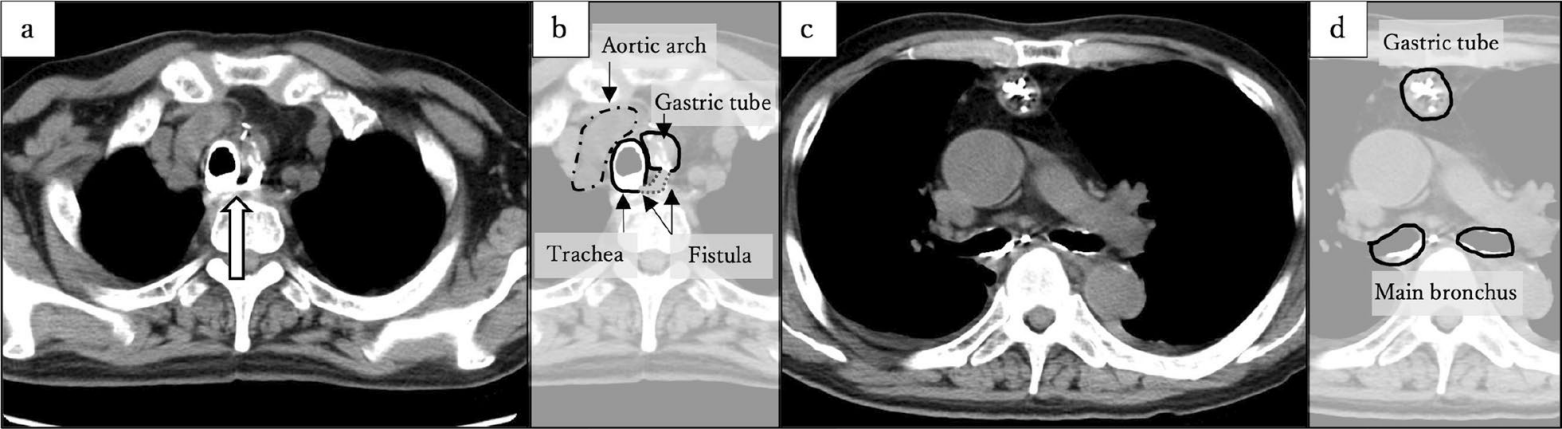
Computed tomography. **a** The fistula (arrow) developed on the membranous portion of the trachea. b The illustration corresponds to a. **c** Contrast medium is present in the trachea and main bronchus. **d** The illustration corresponds to c

He was afebrile and showed improvement on hematological studies; however, on postoperative day 29, he presented with high fever. Esophagography revealed a GTF, and the fistula was observed to be extending from the posterior anastomotic wall to the trachea (Fig. 2a–d). Computed tomography (CT) showed the fistula have developed on the membranous portion of the trachea, and contrast medium used by esophagography could be observed in the trachea and the main bronchus (Fig. 3a–d). The fistula was located higher than the midpoint of the sternal notch and carina.

**Figure. 4:**
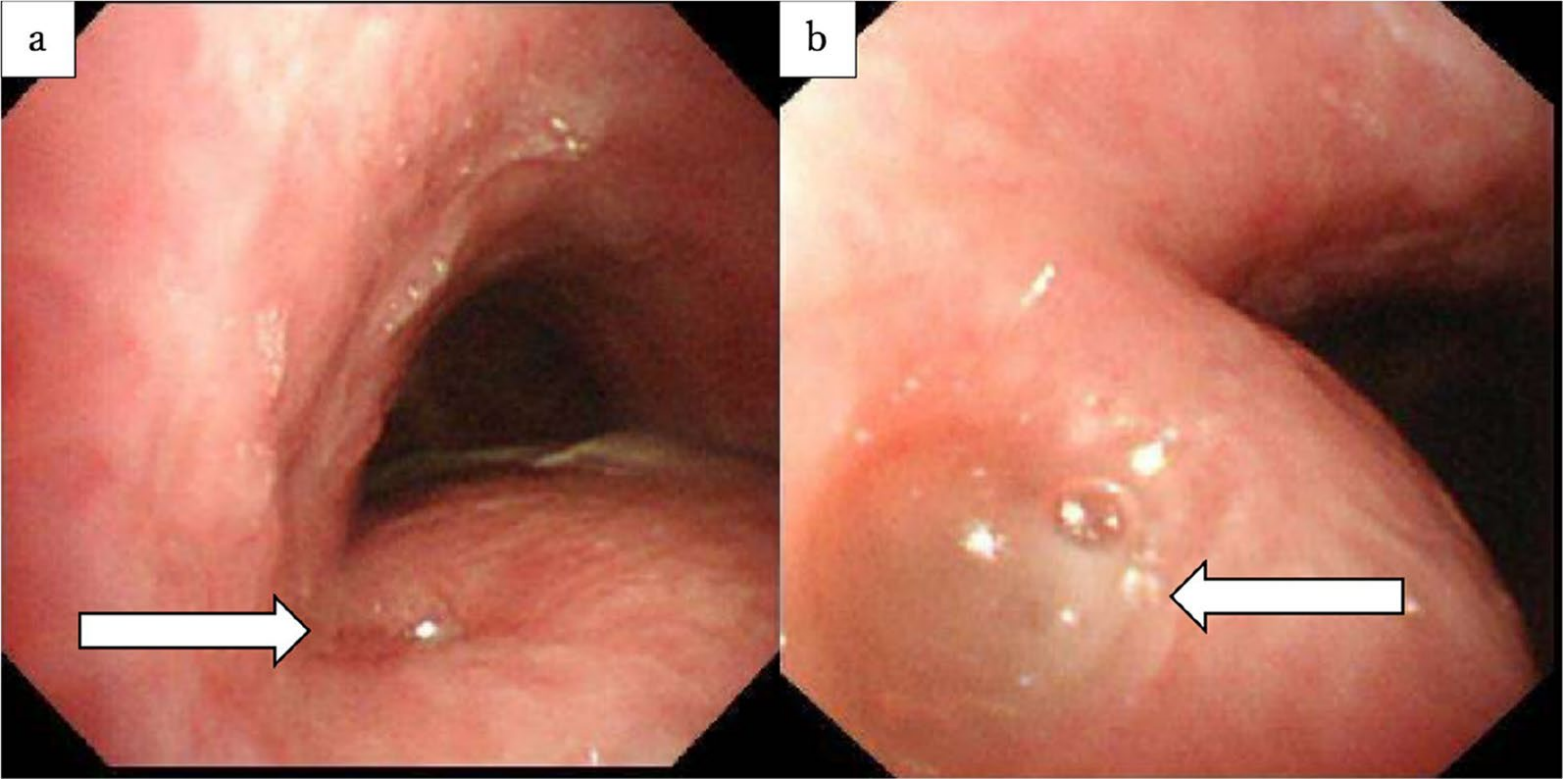
Bronchoscopy. **a**, **b** A hole and bubbling (arrow) can be seen at a GTF on the left side of the membranous portion

**Figure. 5:**
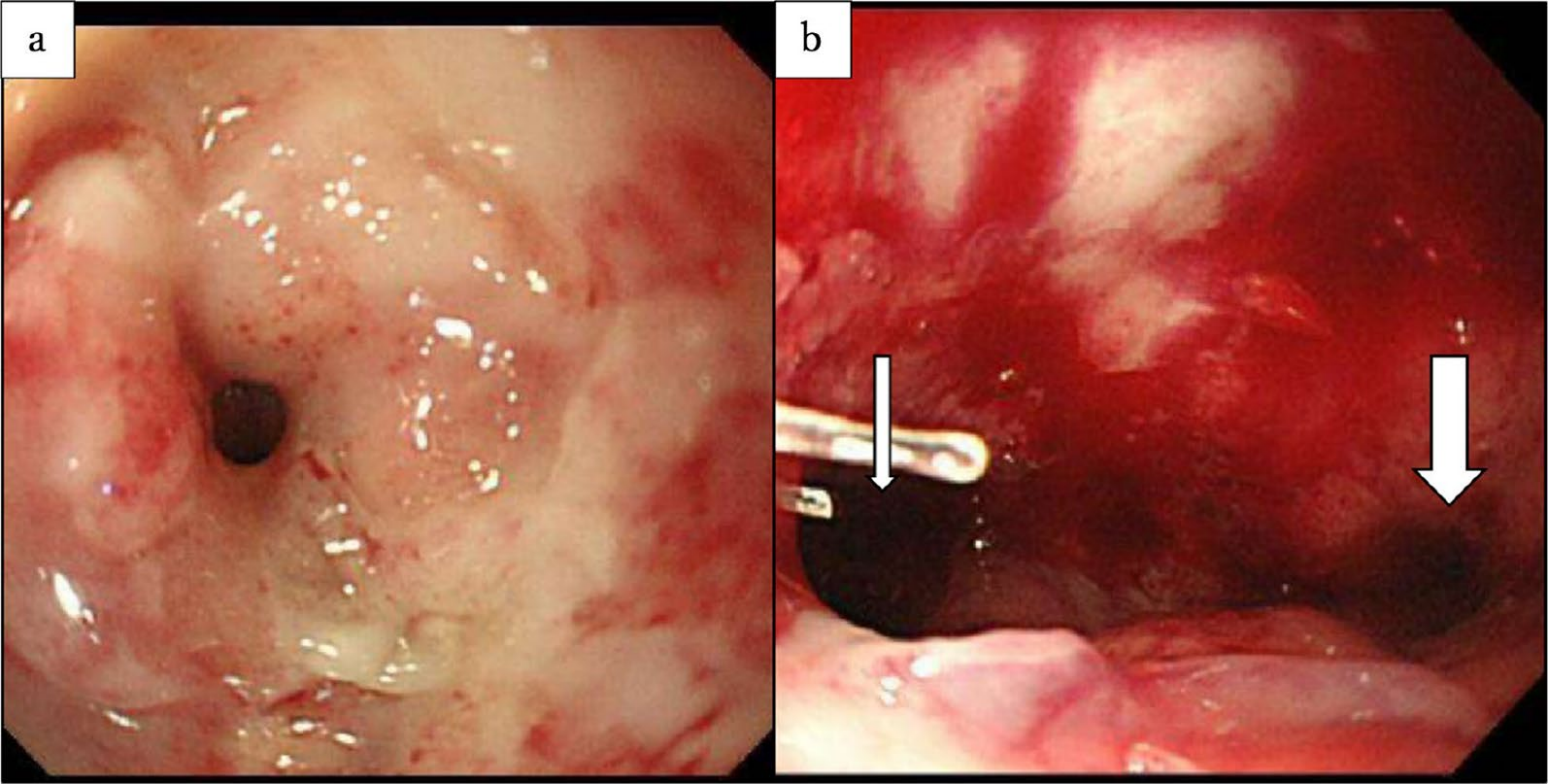
Upper gastrointestinal endoscopy. **a** Anastomotic stenosis was caused by ischemia. **b** After balloon dilatation, a fistula (thin arrow) and gastric tube (thick arrow) can be seen

Bronchoscopy revealed a hole, along with bubbling at the GTF on the left side of the membranous portion (Fig. 4). Upper gastrointestinal endoscopy showed a fistula and anastomotic stenosis caused by ischemia (Fig. 5). Balloon dilatation was performed, and the nasogastric tube was moved to the anastomosis.

On postoperative day 37, upper gastrointestinal endoscopy showed re-constriction of the anastomosis; hence balloon dilatation was repeated. On postoperative day 48, CT and esophagography confirmed that the fistula was undetectable. The patient could intake fluids orally. On postoperative day 56, gastric tube decompression was complete. On postoperative day 60, the patient was able to begin an oral diet. On postoperative day 79, he was transferred to a different hospital for rehabilitation.

## Discussion

We encountered a patient with GTF after esophageal surgery through the retrosternal route, who could be successfully treated with conservative therapy. Here, we identified two important clinical issues: diagnosis of GTF after esophageal surgery through the retrosternal route and successful treatment with only conservative therapy.

GTF after esophageal surgery through the retrosternal route is extremely rare. Most previous reports of GTF describe the occurrence of the fistula through the posterior mediastinal route. Some previous reports described that GTF occurred only in posterior mediastinal route reconstruction [1]. However, in our patient, GTF occurred during retrosternal route reconstruction. So far, only one case of GTF after esophageal surgery through the retrosternal route has been reported [2]. In the case, surgical treatment of GTF was finally needed. Our patient was the first case that conservative treatment was successful for GTF after esophageal surgery through retrosternal route (Table 1).

**Table 1:** GTF cases after esophageal surgery through retrosternal route

	Reference [2]	This case
Age	65	70
Gender	Male	Male
Type of GTF	Necrosis and compression	Leakage
Anastomotic site	Neck	Neck
Radiation	41.4 Gy (preoperative)	None
Site of fistula	High trachea	High trachea
Treatment	Surgical division of the fistula	Conservative
Outcome	Recovered	Recovered

One reason of GTF after esophageal surgery through the retrosternal route is that the treatment for anastomotic leakage was prolonged. The fragile membranous portion of the trachea was perforated because of continuous exposure to the abscess. One case report described a patient diagnosed with pharyngeal abscess, subsequent descending necrotizing mediastinitis, and tracheal ulceration in the membranous portion that eventually perforated [3].

In the case of abscess formation from anastomotic leakage, the options of open drainage, percutaneous drainage, or endoscopic drainage [4] could have been considered. However, open drainage was not performed because the leakage site was on the posterior side of the anastomosis, with contrast pooling in the mediastinum, making drainage more difficult compared to a ventral anastomotic leak. Additionally, the mediastinum tends to become localized due to surrounding tissues, and the patient's general condition was stable at the time.

Similarly, percutaneous drainage was not performed due to the anatomical difficulty of reaching the posterior side of the anastomosis, as it was surrounded by the sternum and the aortic arch, as shown in Fig. 3. Endoscopic drainage was also avoided because it was only 10 days after surgery, and there was concern about stressing the anastomosis. The cavity gradually reduced over time with nasogastric decompression, and the patient’s condition remained stable, so drainage was not pursued.

For these reasons, we chose conservative management with nasogastric decompression alone. Conservative treatment has also been reported for mediastinal abscesses in cases with stable vitals, minimal signs of infection, and spontaneous, effective drainage [5]. However, we acknowledge that earlier drainage could have potentially prevented the GTF.

Another reason could be difficulty in the flow of digestive juices into the gastric tube due to anastomotic stenosis, causing abscess worsening. We believe that balloon dilatation significantly contributed to the treatment outcome.

Few case reports have discussed conservative, endoscopic, and surgical treatment for GTF, with a lack of standard management for GTF. Endoscopic treatment for GTF consists of stent insertion into the gastric tube and/or tracheobronchial tree, clip application, and fibrin glue injection [6, 7]. Surgical treatment of GTF includes resection of the perforated gastric conduit, closure of the bronchial fistula using a pectoralis muscle flap, latissimus dorsi myocutaneous flap or thymus pedicle flap, and reconstruction of the esophagostomy [2, 8–10].

Although surgical treatment shows high recovery rates, it is highly invasive [6]. In contrast, conservative and endoscopic treatments are less invasive and can improve the condition of GTF patients who are otherwise in stable condition.

One previous report summarized 70 cases of GTF, which were subdivided into five types: necrosis type, leakage type, ulcer type, compression type, and other types [6]. The site of the fistula determined the GTF type. In the leakage type, the site of the fistula was located at the same level as the anastomotic site, and when present in the higher trachea, the outcomes were better with conservative therapy.

Another report described the clinical characteristics and outcomes of 10 cases of GTF [11]. The leakage type in the higher trachea appeared between postoperative days 8–35 and had better outcomes with conservative therapy. Our patient had the leakage type of GTF, which met the above conditions for conservative therapy.

One case report described the following conditions for conservative treatment as the optimal management course: (1) absence of critical cough upon swallowing, fever, and recurrent pneumonia; (2) no hematological evidence of acute inflammatory reaction; (3) no evidence of gastric tube necrosis; (4) anastomotic leakage type of GTF appearing approximately two weeks after esophagectomy; and (5) the GTF located in higher trachea [12]. Our case met the above criteria, qualifying for conservative management, which was successful.

If the situation permits, conservative therapy may be the optimal management option because of its simplicity and minimal invasiveness.

Additionally, other minimally invasive methods, such as fistula closure using fibrin glue, PGA sheets [13], or histoacryl [14] have also been reported. In cases where the fistula does not change over time, unlike in this case where repeated balloon dilations gradually reduced the fistula, these methods could be considered.

However, surgical intervention is necessary in case of severe symptoms or failure of conservative treatment.

Most previous reports described that GTF was unique to mediastinal route reconstruction, which involved direct communication between the anastomosis site and adjacent trachea or main bronchus. However, as in this case, we found that GTF could also occur during retrosternal route reconstruction. There are two possible ways for GTF to occur in retrosternal route reconstruction. It can occur because the retrosternal route also includes a part of the neck where the gastric tube and trachea meet or because GTF can be formed through a mediastinal abscess consisting of suture failure at the anastomotic site. In this case, the reason was the latter.

## Conclusions

We encountered a patient with GTF after esophageal surgery through the retrosternal route who was successfully treated with conservative therapy. The possibility of GTF should be considered after retrosternal route reconstruction. When a patient’s general condition and pneumonia are in control, GTF after retrosternal route reconstruction may be cured by conservative treatment.

## Data Availability

Data sharing is not applicable to this article, as no datasets were generated or analyzed in the current study.

## Abbreviations

GTF: Gastro-tracheal fistula
CT: Computed tomography
